# Stalled decline in infant mortality among Palestine refugees in the Gaza Strip since 2006

**DOI:** 10.1371/journal.pone.0197314

**Published:** 2018-06-13

**Authors:** Maartje M. van den Berg, Ali Khader, Majed Hababeh, Wafa’a Zeidan, Silvia Pivetta, Mariam Abd El-Kader, Ghada al-Jadba, Akihiro Seita

**Affiliations:** 1 Department of Paediatrics, Haaglanden Medical Centre, The Hague, the Netherlands; 2 Health Department, Headquarters, United Nations Relief and Works Agency for Palestine Refugees in the Near East (UNRWA), Amman, Jordan; 3 Office for West Bank and Gaza, World Health Organization, Jerusalem, Israel; 4 Health Department, Field Office Gaza, UNRWA, Gaza City, Palestinian Territory; Queensland University of Technology, AUSTRALIA

## Abstract

**Background:**

The United Nations Relief and Works Agency for Palestine refugees in the Near East (UNRWA) has periodically estimated infant mortality rates (IMR) among Palestine refugees in the Gaza Strip (Gaza). These surveys have recorded a decline from 127 per 1000 live births in 1960 to 20.2 in 2006. Thereafter, a survey revealed an IMR of 22.4 in 2011. Alerted by these findings, a follow up survey was conducted in 2015 to further assess the trend of IMR.

**Methods:**

We used the same preceding-birth technique as in previous surveys to estimate IMR and neonatal mortality rate (NMR) per 1000 live births. All multiparous mothers who came to the 22 UNRWA health centers to register their last-born child for immunization were asked if their preceding child was alive or dead. We based our target sample size on the previous IMR of 22.4 and we interviewed 3126 mothers from September to November 2015.

**Findings:**

The third survey estimated mortality rates in 2013. The IMR was 22.7 (95% CI 17.2–28.1) per 1000 live births. IMR did not decline since the estimated IMR of 20.2 (15.3–25.1) per 1000 live births in 2006 and 22.4 (16.4–28.3) per 1000 live births in 2011. NMR was 16.1 (11.6–20.7) per 1000 live births, which was not statistically significantly different from 2006 (12.1 (8.7–16.4)), and was lower than in 2011 (20.3 (15.3–26.2)).

**Conclusion:**

The estimated mortality rate in infants of Palestine refugees in Gaza has not declined since 2006. The stagnation of infant mortality rates indicates that further efforts are needed to investigate causes for this stagnation and ways of addressing the potentially preventable causes among Palestine refugee children in Gaza.

## Introduction

**Fig 1:**
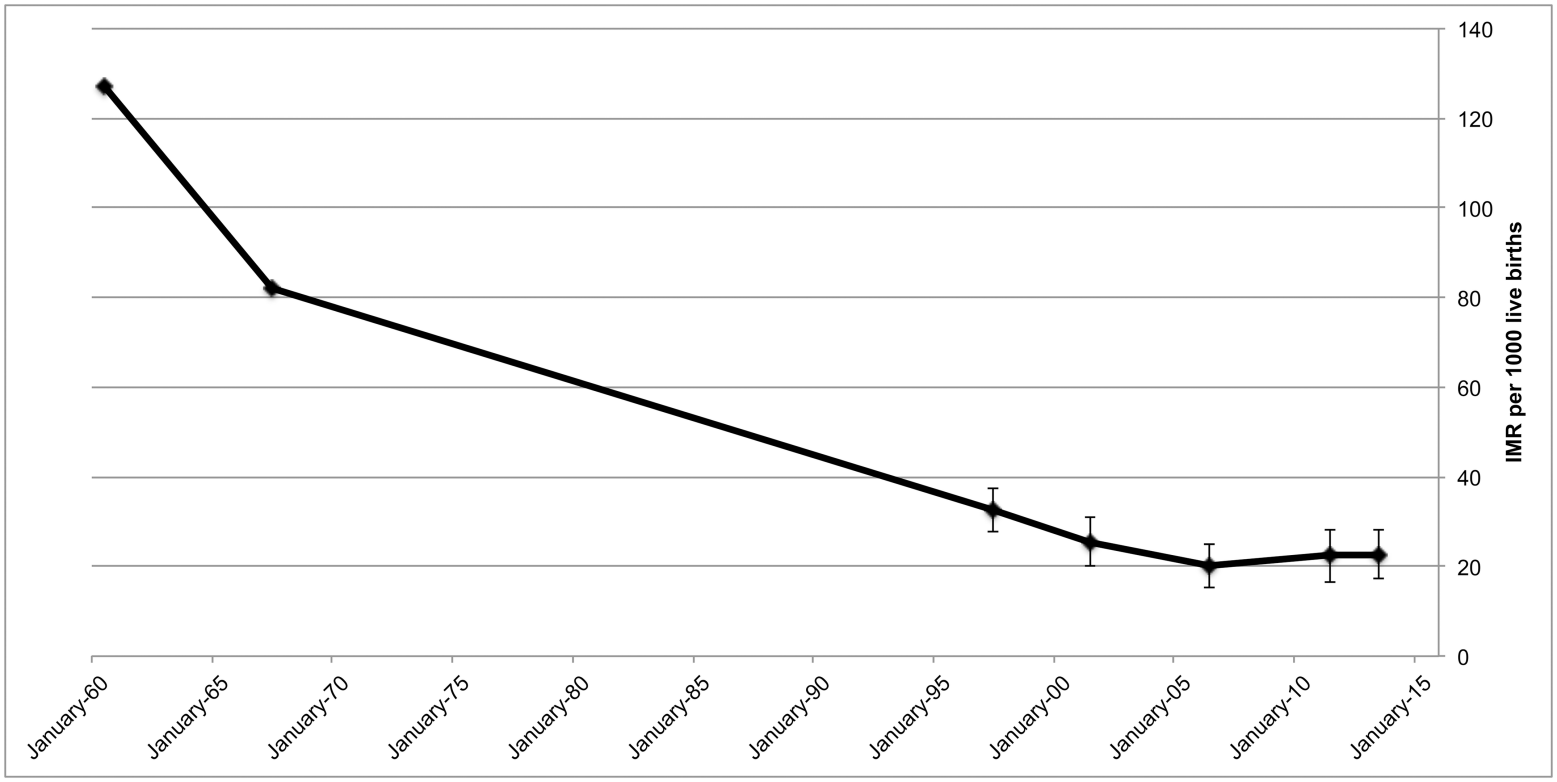
Trend of infant mortality rates among Palestine refugees in Gaza since 1960. The preceding birth-technique was used in 1997, 2003, 2008, 2013 and 2015 surveys, with reference periods from 1995, 2001, 2006, 2011 and 2013 respectively.[1–4] In 1960 and 1967 a different methodology was used. Sources UNRWA surveys.

The United Nations Relief and Works Agency for Palestine refugees in the Near East (UNRWA) is the main Primary Health Care provider to Palestine refugees and has periodically estimated infant mortality rates among Palestine refugees in Gaza. The UNRWA surveys have recorded a decline from 127 per 1000 in 1960, to 82 per thousand in 1967, to 33 per thousand in 1997, to 20.2 per thousand in 2006 (Fig 1).[1–3] The survey with a reference year of 2011 found stagnant infant mortality and an increasing NMR compared to 2006.[4]

The United Nations Millennium Development Goal 4 (MDG4) target of reducing under-five child mortality by two thirds from 1990 to 2015 was successful in many parts of the world.[5,6] However, our previous findings, show that Gaza did not meet that target.[4] Various interventions related to health care provided to mothers and children are effective in reducing child mortality, such as immunizations, provision of skilled birth attendants, and care delivered to newborns directly after birth. Scaling up interventions related to the prevention of preterm births, and the health care for ill and small newborns has been identified as a great obstacle in reducing child mortality.[7] The Sustainable Development Goals (SDG) targets were developed in 2015 with special focus on health care for newborns.[8] By 2030, neonatal deaths should be prevented and all countries should aim to reduce neonatal mortality to 12 per 1000 live births or less.[8] Therefore it needs to be monitored whether strategies for improving neonatal outcome are effective in reducing IMR and NMR trends in Gaza.

As a follow-up to previous UNRWA estimates, we have expanded our series with a survey in 2015. Our first paper focused on differences in IMR and NMR over time and on potential risk factors for infant death and preterm birth. The main purpose of our current paper was to assess again whether IMR and NMR had changed over time in Gaza by using the preceding birth technique. A secondary objective was to verify the reported data about infant deaths with use of hospital records and death certificates. Our previous paper showed that preterm birth, high-risk pregnancies and consanguinity were potential risk factors for infant death.[4] In the current paper we report our assessment of which factors helped to explain differences in the IMR and NMR between the two most recent surveys.

## Methodology

### Preceding birth technique

To ensure comparability with previous data, the preceding-birth technique was used [2–4,9] to estimate infant mortality (the probability of dying between birth and the first birthday) per 1000 live births. Mothers were interviewed and asked if their preceding child was alive or dead. For details of preceding birth technique we refer to our previous publication.[4]

No appropriate registration exists for infant deaths among Palestine refugees in Gaza. Household surveys are unfeasible as many Palestine refugees live outside refugee camps and are therefore difficult to target.[10] The preceding-birth technique is an alternative to prospective analysis and household surveys.[9]

### Study population

The target population included multiparous mothers with at least two children born alive, who attended one of the 22 UNRWA health centers in Gaza for registration and immunization of their most recently-born child. Mothers with only one child and mothers whose most recently-born child was a stillbirth were excluded from this study as the preceding-birth technique concerns the survival or mortality of children born alive. If the preceding pregnancy resulted in miscarriage, information was obtained on the child born before the miscarriage. When the preceding child was born abroad, the child was excluded from analysis.

The following assumptions and requirements were used to calculate the sample size by the Epi-info StatCalc program for population surveys: based on previous data, the estimated infant mortality rate in Gaza was 22.4.[4] A 95% confidence interval of ± 5 deaths per 1000 live births was desired. The estimated sample size needed was 3126 mothers, and was stratified in proportion to the number of registered newborn infants per year at each health center. Enrolment of respondents began on 1 September 2015 and ended on 17 November 2015 when the desired sample size had been reached. The response rate was 98%.

### Data collection

This paper reflects data from 3 surveys. The first survey was performed in 2008 [2], the second survey was conducted in 2013 [4], and for the most recent survey data was collected in 2015. In all surveys a standardized questionnaire was used to collect the required information through face-to-face interviews of mothers, conducted by trained, experienced nurses. For surveys 2 and 3, an identical questionnaire was used, with additional variables which were included after survey 1. The questionnaire included demographic and social variables. Child health records and antenatal records were used to supplement the information provided by the interviewees (S1 Appendix). Pregnancies of the preceding child were categorized by medical staff at the last antenatal visit before delivery as: normal (no risk factor), alert-risk (one risk factor), or high-risk (2 or more risk factors). Examples of risk factors are pregnancy-induced hypertension, or symptoms of pre-eclampsia, gestational diabetes mellitus or anaemia.

In collaboration with the World Health Organization and health authorities in Gaza a thorough review of data on infant deaths was performed after initial data collection, in order to validate the total number of deaths and to get better insight in the causes of death. A modified verbal autopsy questionnaire was used to collect data from medical records and death certificates. The verbal autopsy questionnaire was based on the shortened Population Health Metrics Research Consortium and aimed at determining causes of deaths.[11] This tool included questions about age at death, date of death and signs observed in the child prior to death. For neonatal deaths, questions on whether the baby was born alive or stillborn, the maternal history, delivery and immediate post-delivery practices were included. Three hospital-based senior medical staff (a midwife, an obstetrician and a pediatrician) conducted the review of the medical records and death certificates.

### Statistical analysis

SPSS version 22.0 was used for statistical analysis. Differences between study populations of second and third survey were assessed by Chi-square or T-test. The mean (SD) birth-interval between the preceding child and the most recently-born child was calculated. This interval was used to determine the period to which the mortality data refer. The reference time of the survey depends on the mean birth-interval and the mean age at registration of the most recently-born infant.[2]

The mortality rate was estimated by dividing the number of dead preceding children by the total number of preceding children (including twins and triplets) born alive. Mothers of whom the most recently-born child died would not attend the clinic and were not included in the survey. Similar to previous surveys and as described by Hill and Aguirre, the estimated mortality rates are adjusted by a correction factor of 1.09, to account for mothers who lost their current child in addition to the mothers who lost both the current and the preceding child.[3,4,9]

Six logistic regression models were used to assess what variables, including the reference period, explained any differences between the mortality rates. The first three models used neonatal death as the dependent variable. The first model was univariate with the survey category (survey 1, 2 and 3) as independent variable. For the second model the survey category (survey 2 and 3) was the independent variable. For the third model we used survey category (survey 2 and 3) as independent variable and it was adjusted for gravida, maternal age, maternal education, consanguinity, preterm birth, low birth weight, twins, pregnancy risk classification and birth interval. All independent variables were tested for confounding and interaction. Selection of the relevant variables was based on the assessment of the adjustment effect for each separate variable on survey category with infant death as the dependent variable. If the regression coefficient changed more than 10% with adjustment the variable was selected as relevant confounder and included in the final model. No significant interactions between variables were identified. The 4^th^ to 6^th^ model used infant death as the dependent variable and similar independent variables and confounding factors were used as for neonatal death.

### Ethical considerations

Informed consent from participants was obtained verbally. After verbal explanation of the purpose of the research and the content of the interview, participants’ consent was documented on the data collection sheet. The Ethics Office of United Nations Relief and Works Agency at Headquarters in Amman, Jordan, approved the research proposal and the consent procedure.

## Results

Table 1 shows the demographics of the study population of the three most recent surveys. The populations vary among surveys. The mothers that were interviewed in survey 3 were older, had higher education, lower gravida, lived less frequently in a camp, and less frequently had a consanguineous marriage compared to the mothers studied in survey 2. Also, in survey 3 more mothers had a normal pregnancy (without risk factors) of the preceding child. In addition, the mean birth interval varied among the studies. The occurrence of preterm birth was comparable among studies. Although the difference in age, education and gravida were statistically significantly, the absolute differences may not be practically relevant (Table 1).

**Table 1:** Descriptive data of the study populations for each survey.

Survey		1	2	3
**Newborn under registration**		**n = 3706**	**n = 3128**	**n = 3123**
Sex–male		1895 (51%)	1566 (50.1%)	1587 (50.8%)
		49.5%–52.7%	48.3%–51.9%	49.1%–52.6%
Age at registration in days		8.1 (±5.2)	8.7 (±6.5)	8.6 (±7.0)
**Mother**		**n = 3706**	**n = 3128**	**n = 3123**
Age in years		28.5 (±5.9)	27.8 (±5.5)	28.6 (±5.5)*
Education in years		11.5 (±3.2)	12.6 (±3.1)	12.8 (±3.0)#
Number of pregnancies		5.1 (±2.8)	4.6 (±2.6)	4.4 (±2.4)#
Living inside camp		no data	1038 (33.4%)	940 (30.1%)#
			31.8%–34.9%	28.5%–31.7%
Working mothers		no data	287 (9.2%)	310 (9.9%)
			8.2%–10.4%	8.9%–11.0%
Consanguineous marriage		no data	960 (30.7%)	822 (26.2%)*
			29.1%–32.4%	24.7%–27.7%
Risk classification preceding pregnancy	normal^	no data	2014 (64.4%)	2230 (71.4%)*
			62.7%–66.0%	69.8%–73.0%
	Alert	no data	657 (21.1%)	557 (17.8%)
			19.7%–22.5%	16.5%–19.1%
	High	no data	449 (14.4%)	334 (10.7%)
			13.2%–15.7%	9.6%–11.8%
**Preceding child**			**n = 3162**	**n = 3166**
Gestational age in weeks		no data	39.3 (±1.7)	39.2 (±1.8)
Birth weight in grams		no data	3262.9 (±528.0)	3243.0 (±528.3)
Preterm birth (< 37 weeks)		no data	157 (5.0%)	176 (5.5%)
			4.2%–5.7%	4.7%–6.3%
Low birth weight (< 2500 gram) and preterm		no data	86 (2.7%)	85 (2.7%)
			2.2%–3.3%	2.1%–3.3%
Low birth weight and full term		no data	90 (2.8%)	88 (2.8%)
			2.2%–3.4%	2.2%–3.4%
Birth interval (months)		36.8	32.8 (±20.8)	34.6 (±19.5)*

Data are presented as mean (±SD) or n (%) with 95% confidence intervals; survey 2 vs survey 3:

^#^p <0.05,

*p <0.001;

^ missing data survey 2: 8 and survey 3: 2.

The third survey showed a mean birth-interval of 34.6 months (±19.5) and a mean age at registration of the most recently-born of 8.6 (±7.0) days. The reference time to which the mortality rates refer is December 2013. Reference times of the two previous studies were January 2006 and November 2011. The mortality rates of the three studies are presented in Fig 1 and Table 2.

**Table 2:** Neonatal and infant mortality compared to previous surveys.

Survey				1	2	3
Reference time of mortality rate				January 2006	November 2011	December 2013
Time since 1st survey (months)					70	95
Live births				3706	3162	3166
Neonatal deaths (≤28 days)				41	59	47*
Post-neonatal deaths (>28–1 year)				28	6	19*
Infant deaths (<1 year)				69	65	66*
**Neonatal mortality rate (95% CI)**				12.1 (8.7–16.4)	20.3 (15.3–26.2)	16.1 (11.6–20.7)
**Model**	1	Neonatal death	OR (95% CI)	reference	1.7 (1.1–2.6)	1.4 (0.9–2.1)
	2	Neonatal death	OR (95% CI)		reference	0.8 (0.5–1.2)
	3	Neonatal death	AOR (95% CI)		reference	0.8 (0.5–1.3)
**Infant mortality rate (95% CI)**				20.2 (15.3–25.1)	22.4 (16.4–28.3)	22.7 (17.2–28.1)
	4	Infant death	OR (95% CI)	reference	1.1 (0.8–1.6)	1.1 (0.8–1.6)
	5	Infant death	OR (95% CI)		reference	1.0 (0.7–1.4)
	6	Infant death	AOR (95% CI)		reference	1.0 (0.7–1.6)

* data before validation of infant deaths;

Hosmer-Lemeshow test in logistic regression models 1, 2, 4 and 5: Chi-square 0.00, p-value 1.00; model 3 Chi-square 10.7, p-value 0.22;, model 6 Chi-square 3.10, p-value 0.93. Model 3 and 6 were adjusted for gravida, maternal age, maternal education, consanguinity, preterm birth, low birth weight, twins, pregnancy risk classification and birth interval.

Hosmer-Lemeshow tests did not show lack of fit of the models (Table 2). Compared to 2006, IMR did not increase in 2011 and 2013. From 2006 to 2011 NMR increased and it remained stagnant thereafter. The reduction of NMR from 2011 to 2013 was not statistically significant. When adjusted for confounding factors IMR and NMR did not change between 2011 and 2013 (Table 2). Potential risk factors for infant death were maternal age of 35 years or older (OR (95% CI) 2.2 (1.0–4.8)), consanguinity (2.4 (1.6–3.5)), preterm birth (5.2 (3.1–8.9)) and low birth weight (6.2 (3.6–10.4)), S1 Table.

Since our data depend on mothers’ recall of timing and cause of death, we collaborated with the World Health Organization and health authorities in Gaza to validate data reported by mothers. Maternal and infant case records were not available in 19/62 (30%) of cases. Data collected during the validation showed that 4 false positive infant death cases were reported. Three cases were identified as stillbirths and one preceding child died abroad. The corrected IMR was 21.3 per 1000 live births, and the corrected NMR was 14.4 per 1000 live births. These rates were not used for comparison with previous surveys since those surveys did not use this correction. No information is available on false negative cases since only mothers who reported infant deaths were interviewed for validation of data.

Among all 62 infant deaths, there were 42 neonatal deaths (68%), and 20 (32%) occurred in the first day of life. The most common immediate causes of death were infection (n = 18, 29%), congenital abnormalities (n = 14, 23%) and birth asphyxia (n = 6, 10%). Prematurity was the immediate cause of two deaths (3%) and a contributing cause of death in 15 (24%) of all infant deaths. One infant died due to injuries after a transportation accident.

## Discussion

In our previous paper we presented a statistically significant rise in the neonatal mortality rate (NMR) among infants of Palestine refugees in Gaza from 12.1 per 1000 live births in 2006 to 20.3 in 2011.[4] There is no evidence from the current study that this rise has persisted. NMR in 2013 was 16.1 (or 14.4 when using corrected data after validation of infant deaths). We can conclude that infant mortality rates among Palestine refugees in Gaza are stagnant since 2006. This finding also needs our attention since the ultimate goal is to maintain a continuing decline of infant mortality and to stop preventable infant deaths. Gaza was not able to meet the MDG4 target of a reduction of under-five child mortality by two thirds.[5] Efforts should be made to achieve the new SDG target of NMR below 12 per 1000 live births in 2030.[8] It is important to further assess what the underlying reasons are for the lack of decline of IMR and NMR.

In Gaza, the socioeconomic situation has deteriorated dramatically in the past decade following the imposition of a blockade by the Israeli government in 2007, with military assaults in June 2006, December 2008 to January 2009, November 2012, and July and August 2014.[12] The blockade affected the health sector in Gaza, as hospitals continue to lack adequate physical infrastructure, drugs, supplies, and infection prevention materials.[1,13] It is reasonable to assume that the unstable power supply, the deteriorating functionality of medical equipment, the periodic shortages of essential drugs and medical consumables have had an impact on the quality of medical care with a consequent impact on infant mortality. Although it is not possible with our data to attribute the stalled decline in infant mortality to the siege, it should be noted that the stalling began at the same time as the siege.

Infant mortality rates in Israel and Gaza have been widely divergent. Infant mortality in Israel fell below 20 per 1000 live births in 1977 (Child Mortality Estimates country data), and most recent estimates show a rate of 3 per 1000 in 2015 (Child Mortality Estimates country data). In the West Bank, IMR is usually slightly lower than in Gaza. In 2014, an IMR of 17 was documented for infants born in the West Bank.[14] In addition, the IMR in Egypt was 18 per 1000 live births in 2015.[15] However, since various methodologies have been used in various populations, it is difficult to compare rates among countries in the region.

By using the same methodology as previous UNRWA surveys, we aimed for consistency in estimating mortality trends. The preceding-birth technique was developed to detect relative changes in mortality rates, as an alternative to measuring absolute levels.[9] However, as previously described, and as determined with the validation of our data, the preceding birth technique has several limitations.[4] The estimates are based on small numbers of deaths, and the confidence intervals are wide. Preceding stillbirths were not included in this survey and some stillbirths had been reported as early neonatal deaths. Mothers’ remembrance of the exact age at death could be inaccurate. Therefore, the last survey was adapted by asking the date at death rather than age. In addition, timing and age of death that was reported by mothers was verified by a review of hospital records and death certificates, which made this survey less prone to recall bias than previous surveys. Another limitation of the preceding birth technique is that mothers with only one child were not included in this study, which could have resulted in underestimation of infant mortality as primiparous women are more likely to experience perinatal mortality.[16] However, we used the same methodology that was used in previous surveys and there will only be a significant bias if a large proportion of mothers stop childbearing after a single birth; this is not common in Gaza, where the fertility rate is around five births per woman.[17]

Together with the World Health Organisation, UNICEF and health authorities, UNRWA started working on establishing prospective registration of infant births and deaths in Gaza in 2013. In February 2017, UNRWA completed the introduction of electronic medical records in all of its health centers in Gaza.[18] Therefore, the follow-up of all registered pregnancies and births should become easier from now on.

The NMR data were more variable than the IMR data, as there was a marked increase in the second cohort and stagnation thereafter. Another drawback of the study is that confounding variables are lacking for the first survey and therefore adjustments could not be made for the differences between first and second survey.

A slowing down of the reduction of infant mortality in Gaza was previously noted to be related to a rise in neonatal mortality.[17] In Gaza, sixty eight per cent of infants deaths occurred during the neonatal period, compared with 60% reported for North Africa and the Middle East.[15] Worldwide progress in reducing child mortality has been faster for under-five and infant mortality than for neonatal mortality.[19,20] The persisting toll of neonatal conditions and congenital malformations on child survival is clear, especially in low-income and low-middle-income countries.[15] The most neonatal deaths worldwide are attributable to three main causes: infections, intra-partum conditions and preterm birth complications.[19] In Palestine refugees in Gaza congenital malformations contributed also frequently to infant death. In order to prevent neural tube defects, preconception care was integrated in UNRWA’s maternal and child health program in 2011 [18], and includes daily folic acid supplementation before pregnancy and in the first trimester to prevent neural tube defects.

Among the pregnant Palestine refugee women, almost all (95%) have at least four antenatal visits during pregnancy, with an average of 6.7 visits.[18] It is important to further assess how death due to birth defects can be prevented in Gaza. Potentially, antenatal screening for congenital malformations by routine antenatal ultrasonography could play a role in this. Such screening is currently not standard practice in UNRWA health centers.

Immunization to prevent infection related infant deaths is provided to almost 100% of the infants registered at UNRWA in Gaza.[18] Infection prevention and control practices including hand hygiene are effective in reducing infant deaths.[21] In Gaza there is room for improvement on hand hygiene practices [22] in the hospitals and among the community. Development and implementation of infection prevention programs are required for the reduction of infant deaths.

UNRWA continues to work with its partners on improving neonatal survival in order to achieve the SDG target in 2030. In our previous cohort, we identified consanguinity, preterm birth and high-risk pregnancy as potential risk factors for infant death.[4] Current survey identified again consanguinity and preterm birth as potential risk factors, in addition to older maternal age and low birth weight. Although in the third survey, the prevalence of consanguinity and high-risk pregnancies were lower, IMR remained stagnant. The occurrence of preterm birth did not vary between the last two cohorts. The UNRWA health program already promotes the positive effects achievable with family planning services and continues to improve antenatal care for women with high-risk pregnancies. In addition, enhancing public awareness on the negative health consequences of consanguinity, which increases the risks of congenital malformations, is required.

In conclusion, the mortality rate among infants of Palestine refugees in Gaza has not shown a decline since 2006. The rise of neonatal mortality in the previous survey was not confirmed. Stagnation of infant and neonatal mortality rates indicates that further investigation is needed to understand how the stalled decline in IMR can be addressed. Efforts are needed to reduce future infant deaths. Besides optimizing the prevention of preterm births, infections and congenital malformations, improving quality of care during childbirth and during the early neonatal period will be crucial to reduce neonatal and thereby infant mortality. Nevertheless, in the absence of a healthier political and socioeconomic situation, and with recent drop in financial funding for UNRWA [23], it is likely to remain challenging to reduce infant mortality in Gaza.

## Supporting information

S1 Appendix Data collected during interview and by review of maternal and child health records. (PDF)

S1 Table Potential risk factors for infant death. (PDF)

## References

[1] Giacaman R, Khatib R, Shabaneh L, Ramlawi A, Sabri B, Sabatinelli G, et al Health status and health services in the occupied Palestinian territory. Lancet. 2009;373:837–49.

[2] Riccardo F, Khader A, Sabatinelli G. Low infant mortality among Palestine refugees despite the odds. Bull World Health Organ. 2011;89(4):304–11. doi: 10.2471/BLT.10.082743 21479095 10.2471/BLT.10.082743 PMC3066522

[3] Madi H. Infant and child mortality rates among Palestinian refugee populations. Lancet. 2000;356:2000. 10.1016/S0140-6736(00)02511-3 11071191

[4] van den Berg MM, Madi HH, Khader A, Hababeh M, Zeidan W, Wesley H, et al Increasing Neonatal Mortality among Palestine Refugees in the Gaza Strip. PLoS One. 2015;10(8):e0135092 doi: 10.1371/journal.pone.0135092 26241479 10.1371/journal.pone.0135092 PMC4524592

[5] Resolution adopted by the General Assembly—United Nations Millennium Declaration (55/2). New York; 2000.

[6] Wang H, Liddell CA, Coates MM, Mooney MD, Levitz CE, Schumacher AE, et al Global, regional, and national levels of neonatal, infant, and under-5 mortality during 1990–2013: a systematic analysis for the Global Burden of Disease Study 2013. Lancet. 2014;384:957–79. doi: 10.1016/S0140-6736(14)60497-9 24797572 10.1016/S0140-6736(14)60497-9 PMC4165626

[7] Bhutta ZA., Das JK, Bahl R, Lawn JE, Salam RA., Paul VK, et al Can available interventions end preventable deaths in mothers, newborn babies, and stillbirths, and at what cost? Lancet. 2014;384:347–70. doi: 10.1016/S0140-6736(14)60792-3 24853604 10.1016/S0140-6736(14)60792-3

[8] United Nations General Assembly. UN Sustainable Development Goals. http://www.un.org/ Sustain. 2015

[9] Hill AG, Aguirre A. Childhood Mortality Estimates using the Preceding Birth Technique : Some Applications and Extensions. Popul Stud (NY). 1990;44:317–40.

[10] UNRWA Health Department. Annual report 2012. Amman, Jordan; 2013.

[11] Serina P, Riley I, Stewart A, Flaxman AD, Lozano R, Mooney MD, et al A shortened verbal autopsy instrument for use in routine mortality surveillance systems. BMC Medicine. 2015;13:302 doi: 10.1186/s12916-015-0528-8 26670275 10.1186/s12916-015-0528-8 PMC4681088

[12] UNRWA. Gaza in 2020—unrwa operational response. Gaza; 2013.

[13] Gilbert M. Brief report to UNRWA : The Gaza Health Sector as of June 2014. Tromso, Normway; 2014.

[14] PCBS, MICS, UNICEF U. Palestinian Multiple Indicator Cluster Survey 2014. 2015.

[15] GBD 2015 Child Mortality Collaborators. Global, regional, national, and selected subnational levels of stillbirths, neonatal, infant, and under-5 mortality, 1980–2015: a systematic analysis for the Global Burden of Disease Study 2015. Lancet. 2016;388:1725–74. doi: 10.1016/S0140-6736(16)31575-6 27733285 10.1016/S0140-6736(16)31575-6 PMC5224696

[16] Aminu M, Unkels R, Mdegela M, Utz B, Adaji S, van den Broek N. Causes of and factors associated with stillbirth in low- and middle-income countries: a systematic literature review. BJOG An Int J Obstet Gynaecol. 2014;121:141–53. 10.1111/1471-0528.12995 25236649

[17] Rahim HFA, Wick L, Halileh S, Hassan-Bitar S, Chekir H, Watt G, et al Maternal and child health in the occupied Palestinian territory. Lancet. 2009;373(9667):967–77. doi: 10.1016/S0140-6736(09)60108-2 19268353 10.1016/S0140-6736(09)60108-2

[18] UNRWA Health Department. UNRWA Annual Report 2016. Amman, Jordan; 2016.

[19] Lawn JE, Blencowe H, Oza S, You D, Lee ACC, Waiswa P, et al Every newborn: Progress, priorities, and potential beyond survival. Lancet. 2014;384:189–205. doi: 10.1016/S0140-6736(14)60496-7 24853593 10.1016/S0140-6736(14)60496-7

[20] Lozano R, Wang H, Foreman KJ, Rajaratnam JK, Naghavi M, Marcus JR, et al Progress towards Millennium Development Goals 4 and 5 on maternal and child mortality: an updated systematic analysis. Lancet. 2011;378(9797):1139–65. doi: 10.1016/S0140-6736(11)61337-8 21937100 10.1016/S0140-6736(11)61337-8

[21] Graham PL. Simple strategies to reduce healthcare associated infections in the neonatal intensive care unit: line, tube, and hand hygiene. Clin Perinatol. 2010;37:645–53. doi: 10.1016/j.clp.2010.06.005 20813276 10.1016/j.clp.2010.06.005

[22] Al-Khatib IA; Abusara LW; Odeh YM; Sbeih SA; Massoud MA. Hand washing among Palestinians in the West Bank and Gaza Strip: attitudes and practices. J Env Heal. 2015;77:50–6. 25619036

[23] Seita A, Goldsmith A, Hababeh M, Shahin Y. Amid US funding cuts, UNRWA appeals for health and dignity of Palestinian refugees. Lancet. 2018;6736:18–9. 10.1016/S0140-6736(18)30113-2 29395267

