# Supplementary material for: Stalled decline in infant mortality among Palestine refugees in the Gaza Strip since 2006
Source: PLoS One. 2018 Jun 13;13(6):e0197314. doi: 10.1371/journal.pone.0197314 (PMC5999100; doi:10.1371/journal.pone.0197314)
Supplement: S1 Appendix — (PDF) [file pone.0197314.s001.pdf]

S1 Appendix. Data collected during interview and by review of maternal and child health records.

	Question	Item	Present Infant Child Health Record	Maternal Antenal Record	Preceding child Child Health Record	Interview
Information about present child	q-1	S/No				
	q-2	Field	√			
	q-3	Child Health Record No	√			
	q-4	Health Center	√			
	q-5	Name of interviewer				
	q-6	Verbal informed consent				√
	q-7	Gender present child	√			
	q-8	Date of registration (dd-mm-yyy)	√			
	q-9	Date of Birth (dd-mm-yyy)	√			
Information about the mother	q-10	Mother's Date of Birth		√		
	q-11	Total No. of living children - Male				√
		Total No. of living children - Female				√
	q-12	Total number of pregnancies "Gravida"		√		
	q-13	Consanguinity		√		
	q-14	Mother's education (in years)		√		
	q-15	Mother employed		√		
	q-16	Residence		√		
Information about the preceding child	q-17	Preceding child single/ twins/ triplet		√	√	√
	q-18	Gender preceding child		√	√	√
	q-19	Date of Birth preceding child (dd-mm-yyy)		√	√	√
	q-20	Pregnancy risk of the preceding child		√		√
	q-21	Gestational age (in weeks)		√		√
	q-22	Birth Weight (in gram)		√	√	√
	q-23	Condition of Preceding Child (Alive / Dead)			√	√
Information about the preceding	q-24	Age of Death of Preceding Child			√	√
	q-25	Place of Death			√	√
	q-26	Cause of death			√	√
