# Supplementary material for: Stalled decline in infant mortality among Palestine refugees in the Gaza Strip since 2006
Source: PLoS One. 2018 Jun 13;13(6):e0197314. doi: 10.1371/journal.pone.0197314 (PMC5999100; doi:10.1371/journal.pone.0197314)
Supplement: S1 Table — (PDF) [file pone.0197314.s002.pdf]

S1 Table. Potential risk factors for infant death.

Independent variables		Infant death<br>*Adjusted odds ratio (95% CI)*
**Reference period**	2011	reference
	2013	1.1 (0.7 - 1.6)
**Age of mother at preceding birth**	18-34 years	reference
	< 18 years	0.6 (0.2 - 1.6)
	≥ 35 years	2.2 (1.0 - 4.8)
**Maternal education – less than 12 years**		1.5 (1.0 - 2.2)
**Number of pregnancies**	≤ 3	reference
	4 - 5	1.3 (0.8 - 2.1)
	≥ 6	0.9 (0.5 - 1.6)
**Consanguinity**		2.4 (1.6 - 3.5)
**Risk classification preceding pregnancy**	normal	reference
	alert	1.2 (0.7 - 2.0)
	high	1.5 (0.9 - 2.6)
**Twin/triplet pregnancy**		1.7 (0.8 - 3.9)
**Birth-interval below 24 months**		1.3 (0.9 - 2.0)
**Preterm birth (< 37 weeks)**		5.2 (3.1 - 8.9)
**Low birth weight (< 2500 grams)**		6.2 (3.6 - 10.4)
Hosmer and Lemeshow test		*Chi-square = 3.1, p = 0.93*
